# Cell-extrinsic consequences of epithelial stress: activation of protumorigenic tissue phenotypes

**DOI:** 10.1186/bcr3368

**Published:** 2012-12-07

**Authors:** Colleen A Fordyce, Kelley T Patten, Tim B Fessenden, RosaAnna DeFilippis, E Shelley Hwang, Jianxin Zhao, Thea D Tlsty

**Affiliations:** 1 Department of Pathology, University of California, San Francisco, HSW 501, 513 Parnassus Avenue, San Francisco, CA 94143, USA; 2 Cancer Biology and Institute for Biophysical Dynamics, University of Chicago, GCIS W309, 929 E 57th Street, Chicago, IL 60642, USA; 3 Department of Surgery, Duke University, Seeley Mudd Building Room 465, Duke University Medical Center, Durham, NC 27710, USA

## Abstract

**Introduction:**

Tumors are characterized by alterations in the epithelial and stromal compartments, which both contribute to tumor promotion. However, where, when, and how the tumor stroma develops is still poorly understood. We previously demonstrated that DNA damage or telomere malfunction induces an activin A-dependent epithelial stress response that activates cell-intrinsic and cell-extrinsic consequences in mortal, nontumorigenic human mammary epithelial cells (HMECs and vHMECs). Here we show that this epithelial stress response also induces protumorigenic phenotypes in neighboring primary fibroblasts, recapitulating many of the characteristics associated with formation of the tumor stroma (for example, desmoplasia).

**Methods:**

The contribution of extrinsic and intrinsic DNA damage to acquisition of desmoplastic phenotypes was investigated in primary human mammary fibroblasts (HMFs) co-cultured with vHMECs with telomere malfunction (TRF2-vHMEC) or in HMFs directly treated with DNA-damaging agents, respectively. Fibroblast reprogramming was assessed by monitoring increases in levels of selected protumorigenic molecules with quantitative polymerase chain reaction, enzyme-linked immunosorbent assay, and immunocytochemistry. Dependence of the induced phenotypes on activin A was evaluated by addition of exogenous activin A or activin A silencing. *In vitro *findings were validated *in vivo*, in preinvasive ductal carcinoma *in situ *(DCIS) lesions by using immunohistochemistry and telomere-specific fluorescent *in situ *hybridization.

**Results:**

HMFs either cocultured with TRF2-vHMEC or directly exposed to exogenous activin A or PGE_2 _show increased expression of cytokines and growth factors, deposition of extracellular matrix (ECM) proteins, and a shift toward aerobic glycolysis. In turn, these "activated" fibroblasts secrete factors that promote epithelial cell motility. Interestingly, cell-intrinsic DNA damage in HMFs induces some, but not all, of the molecules induced as a consequence of cell-extrinsic DNA damage. The response to cell-extrinsic DNA damage characterized *in vitro *is recapitulated *in vivo *in DCIS lesions, which exhibit telomere loss, heightened DNA damage response, and increased activin A and cyclooxygenase-2 expression. These lesions are surrounded by a stroma characterized by increased expression of α smooth muscle actin and endothelial and immune cell infiltration.

**Conclusions:**

Thus, synergy between stromal and epithelial interactions, even at the initiating stages of carcinogenesis, appears necessary for the acquisition of malignancy and provides novel insights into where, when, and how the tumor stroma develops, allowing new therapeutic strategies.

## Introduction

Cellular responses to stress are complex and vary, depending on the cell type, the extent and type of DNA damage or stress, and the surrounding environment and temporal considerations. Stress can activate a variety of cell-intrinsic processes, including autophagy, the unfolded protein response, and the DNA damage response (DDR), as well as more irreversible phenotypes, such as apoptosis or senescence. Cell-intrinsic consequences of cellular stress often require the initial activation of the DDR through insults including: DNA damage, telomere malfunction, and hypoxic stress [1,2]. The DDR facilitates DNA repair by recruiting and activating DNA-repair proteins in an attempt to maintain genomic integrity. Additionally, the DDR activates p53 and Rb pathway-dependent barriers to malignancy through the induction of cell-cycle arrest, apoptosis, or senescence [1,3]. Compromising these barriers can lead to genomic instability and the acquisition of tumorigenic phenotypes [3-7]. However, cellular-stress responses are also associated with cell-extrinsic phenotypes [5,8,9].

We recently showed that the consequences of DDR in mortal, nontumorigenic human mammary epithelial cells can also be cell extrinsic. These responses are not confined to the initial cell that is stressed, but can also be transmitted to adjacent (nondamaged) epithelial cells through paracrine secretion of stress-induced factors (for example, an activin A-dependent induction of cyclooxygenase 2 (COX-2)) [5]. In the present study, we investigated whether activating DDR in primary human mammary epithelial cells (derived from disease-free tissues), could have cell-extrinsic consequences, resulting in induction of genes associated with protumorigenic phenotypes in adjacent fibroblasts *in vitro*.

It is now well recognized that stromal cells within and surrounding pathologic lesions are not simply passive structural components, but also actively contribute to malignant phenotypes through elevated expression of cytokines and growth factors [10-15]. They exert their effects through increased deposition and remodeling of the ECM, reprogramming of metabolism, local alteration of immune function, and increased vascularization. Collectively, these alterations are known as desmoplasia. Carcinoma-associated fibroblasts (CAFs) are among the predominant cell types in the tumor stroma and contribute to most of the phenotypes described earlier [10,13,16,17]. As expected from these *in vitro *phenotypes, CAFs promote tumorigenesis in animal models of cancer [11,12]. Where, when, and how CAFs come to acquire these properties is under intensive study [10,13-15].

Recent studies suggest that multiple secretory pathways may participate in the development of a protumorigenic stroma [8,9]. In this study, we show that, in addition to reprogramming adjacent epithelial cells, stress-elicited factors from epithelial cells can also reprogram adjacent stromal cells. Additionally, we show that cell-intrinsic DNA damage in human mammary fibroblasts (HMFs) also results in an induction of activin A and an upregulation of genes associated with a tumor stromal program similar, but not identical, to the program elicited by cell-extrinsic signaling. *In vivo*, we demonstrated that preinvasive lesions (ductal carcinoma *in situ*, DCIS) exhibiting a DDR (shorter telomeres and γH2AX foci) are associated with high activin A and a stromal signature consistent with protumorigenic phenotypes. Collectively, these data suggest that DNA damage (telomere malfunction) in nonmalignant epithelial cells has cell-extrinsic consequences for neighboring epithelial and stromal cells and implies that the generation of protumorigenic stromal phenotypes can occur early in tumorigenesis. These studies provide novel targets for the prevention of preinvasive lesions.

## Materials and methods

### Cell culture and induction of DDR

#### Monocultures

Human mammary fibroblasts (HMFs) were isolated from disease-free tissues obtained from six individuals: RM9, RM15, RM21, RM111, RM124, and RM156. HMFs are primary cells with a finite proliferative capacity, and, given the number of end points studied, the same HMFs could not be used for all experiments. However, to ascertain the relevance of the described phenotypes and to account for potential interindividual variations, all experiments were carried out with three independent HMFs obtained from our collection of six individuals listed earlier, unless otherwise stated. As previously described, HMFs were isolated from tissues by using differential centrifugation, filtration, and media selection (RPMI-1640 + 10% fetal bovine serum (FBS)) [11]. Fibroblast identity was confirmed through monitoring expression of specific markers. Unlike MCF7 mammary epithelial cells, HMFs expressed fibronectin, but not E-cadherin, consistent with a fibroblast phenotype (see Additional file 1).

HMFs were incubated for 24 hours in media without serum before the addition of activin A (Sigma-Aldrich, St. Louis MO), PGE_2 _(Cayman Chemicals, Ann Arbor, MI), or the COX-2 inhibitor, NS398 (Cayman Chemicals, Ann Arbor, MI), for 48 hours. Etoposide (Sigma-Aldrich, St. Louis MO), and the DNA-PK inhibitor, NU7026 (Sigma-Aldrich, St. Louis MO), were added to the culture medium (RPMI + 10% FBS) at the doses and times shown in the figure legends. The media were removed before exposure to UVC and immediately replaced. Human mammary epithelial cells with silenced p16^INK4A ^via promoter methylation, referred to as variant human mammary epithelial cells (vHMECs) [7,18], were isolated from disease-free tissues obtained from three women: RM15, RM78, and RM79. vHMECs were propagated in MEGM, as previously described [19]. All experiments were performed on proliferating midpassage cell populations.

#### Co-cultures

*TRF2 *and *hTERT *were overexpressed in vHMECs from RM78 and RM79, as described previously [5]. Transwell dishes (Costar, Tewksbury, MA) with a 0.4-μm pore were used for coculture experiments. In brief, 1.7 × 10^5 ^HMFs from RM15 and RM21 were plated in RPMI + 10% FBS in the bottom chamber. The following day, cells were placed in RPMI without serum for 24 hours. These media were subsequently replaced with MEGM and an equal number of vHMECs from RM78 and RM79 overexpressing either *TRF2, hTERT*, vector (pWP), or mock infected were plated onto the top chamber of the transwell dish. The medium in the top chamber was replaced after 24 hours. HMFs were harvested 48 hours after addition of vHMECs to the transwell dish.

### Cell-wounding assay

HMFs from RM111 and RM124 were plated in RMPI + 5% FBS and were exposed to exogenous activin A (0.08 μg/ml) or vehicle (dH_2_0) for 48 hours. Conditioned media were collected from both HMFs, centrifuged briefly to remove cellular debris, and diluted in MEGM. RPMI + 5% FBS supplemented with either exogenous activin A (0.08 μg/ml) or vehicle was treated identically to the conditioned media. RM15 vHMECs were cultured in a 2:1 mix of MEGM and one of each of the following four media: (a) HMF + activin A-conditioned medium, (b) HMF + vehicle-conditioned medium, (c) RPMI + 5% FBS + activin A, or (d) RPMI + 5% FBS + vehicle. After 24 hours, confluent monolayers of vHMECs were manually disrupted with a pipette tip, and the medium was removed and replaced with MEGM. Duplicate wells for each condition from both HMFs were imaged immediately after wounding and every 4 hours for a total of 28 hours. The size of the "wound" in the vHMEC monolayers was measured in three locations for each condition and time point by using the NIS-Elements D 3.2 software (Nikon).

### Quantitative PCR

Total RNA was isolated from cells and cDNA synthesized by using standard methods. cDNA was subsequently used for quantitative polymerase chain reaction (Q-PCR) by using the standard curve method. Primer-probe sets for each of the genes were obtained from ABI (Table 1). The expression of *GUSB *(IDT), an internal control, was used to normalize for variances in input cDNA. The forward and reverse primer and Taqman probe sequences for *GUSB *were as follows: 5' CTCATTTGGAATTTTGCCGATT 3', 5' CCGAGGAAGATCCCCTTTTTA 3', 5' FAM-TGAACAGTCACCGACGAGAGTGCTGGTA-TAM 3', respectively. Q-PCR was performed on a CFX-96 (Biorad) thermocycler by using the 2x SsoFast Master Mix (Bio-Rad Laboratories).

**Table 1:** Quantitative PCR probes sets

Gene name	Gene abbreviation	ABI catalog
*Activin A (inhibin *β*A)*	INHIB A	Hs00170103_m1

*Prostaglandin-endoperoxide synthase 2*	COX-2	Hs00153133_m1

*Tenascin C*	TenC	Hs01115664_m1

*Fibronectin 1*	FN1	Hs00365052_m1

*Collagen, type I, α 1*	COL1A1	Hs00164004_m1

*Hypoxia-inducible factor 1 α*	HIF1α	Hs00153153_m1

*Lactate dehydrogenase A*	LDHA	Hs00855332_g1

*Interleukin 6*	IL-6	Hs00985639_m1

*Interleukin 8*	IL-8	Hs01567913_g1

*Vascular endothelial growth factor A*	VEGF	Hs00173626_m1

*Signal transducer and activator of transcription 3*	STAT3	Hs01047580_m1

### ELISA and lactate assays

#### ELISAs

Activin A, interleukin 6 (IL-6), interleukin 8 (IL-8), and vascular endothelial growth factor (VEGF) protein levels were measured by using the Duo-Set ELISA kits (R&D Systems, Minneapolis MI). Prostaglandin levels were measured by using the Prostaglandin E_2 _E1A ELISA Kit (Cayman Chemicals, Ann Arbor, MI). To prepare conditioned media, HMFs were plated into duplicate wells of a six-well plate and exposed to each agent or corresponding vehicle controls 24 hours later. Media were replaced the following day and allowed to condition for 48 hours. Conditioned media were collected, centrifuged briefly to remove cellular debris, and stored at -80°C in siliconized tubes.

#### Lactate

Lactate levels were measured in media that were conditioned for 4 hours. In brief, cells were treated as described earlier, and media were replaced with media without exogenous agents. Lactate levels were measured immediately after collection by using Lactate Reagent (Trinity Biotech, Bray, Co Wicklow, Ireland) and compared with a standard curve (Sigma-Aldrich, St. Louis, MO). Expression of each molecule was measured in duplicate assays.

### Immunocytochemistry

The levels of fibronectin and α-smooth muscle actin (αSMA) protein were evaluated in HMFs obtained from RM9, RM15, and RM111. Cells were plated on glass coverslips, grown to confluence, and incubated in media without serum for 24 hours before treatment with activin A for 48 hours. Cells were subsequently washed twice in PBS and then fixed in 4% paraformaldehyde for 20 minutes at room temperature, followed by a graded methanol series. Coverslips were exposed to primary antibodies against fibronectin (1:100, BD Biosciences, San Jose, CA) and αSMA (1:50, Dako, Carpentaria, CA) overnight at 4°C and visualized with secondary antibodies labeled with FITC. Nuclei were counterstained with 4'-6-diamidino-2-phenylindole (DAPI). Tissues were imaged by using a Zeiss LSM 510 confocal microscope with a 20× objective.

### Tissue samples

The 16 cases of nonrecurrent DCIS evaluated in this study were surgically resected at the University of California San Francisco Medical Center from 2008 to 2009. Women treated with neoadjuvant therapy were not included in the study. Clinical and pathologic information was obtained from patients' medical records and pathology reports (Table 2). DCIS lesions were primarily estrogen- and progesterone-receptor positive and grade 2 or 3.

**Table 2:** Patient and tumor characteristics of ductal carcinoma *in situ *(DCIS) cohort

ID	Menopausal status^a^	DCIS grade	ER status	PR status	**Activin A score**^c^	**Telo-FISH**^d^	γH2AX score	COX-2 score-	αSMA score	CD31 score	Immune infiltrate score^e^
96	Post	3	+	+	Low	High	Low	Low	Low	Low	Low

52	Pre/peri	2	+	+	Low	High	Low	Low	Low	Low	Low

13	Post	2	+	+	Low	High	Low	Low	Low	High	Low

19	Post	3	-	-	Low	High	Low	Low	High	Low	Low

50	Pre/peri	1	+	+	Low	High	High	Low	Low	Low	Low

10	Pre/peri	2	+	+	Low	Low	Low	Low	Low	Low	Low

69	Post	3	+	+	Low	ND	Low	Low	Low	Low	High

28	Pre/peri	3	+	Unk^b^	Low	High	High	High	Low	High	High

3	Pre/peri	3	+	+	High	High	High	Low	High	High	High

63	Post	3	-	-	High	ND	High	High	Low	High	High

47	Post	2	+	+	High	ND	High	High	High	High	High

84	Post	3	Unk^b^	Unk	High	Low	High	High	High	Low	High

15	Pre/peri	3	+	+	High	Low	High	High	High	High	High

12	Post	3	-	-	High	Low	High	High	High	High	High

66	Pre/peri	2	+	+	High	Low	High	High	High	High	High

9	Post	3	+	+	High	Low	High	High	High	High	High

**Sum**^f^	Post = 8 Pre/peri = 7	G3 = 10 G2 = 5 G1 = 1	Pos = 12 Neg = 3 Unk = 1	Pos = 11 Neg = 3 Unk = 2	High = 8 Low = 8	High = 7 Low = 6 ND = 3	High = 10 Low = 6	High = 8 Low = 7	High = 8 Low = 8	High = 9 Low = 7	High = 10 Low = 6

^a^Pre- or perimenopausal status (pre/peri) or postmenopausal (post) status was self-reported. ^b^Unknown result (Unk). ^c^Protein expression (activin A, γH2AX, COX-2, αSMA, and CD31) was stratified into two groups, as described in Methods. ^d^Telomere-specific fluorescence *in situ *hybridization (Telo-FISH) intensity was stratified into two groups, as described in Methods. Cases in which telomere intensity could not be determined are shown (ND). ^e^Degree of immune cell infiltrate was evaluated on H&E slides and stratified into two groups, as described in Methods. ^f^Summary of cohort showing the number of cases in each category (ethnicity, mean age at diagnosis, menopausal status, DCIS grade, ER and PR status, Activin A, γH2AX, COX-2, αSMA, and CD31 protein expression and immune infiltration).

All tissues used in this work were accrued with informed patient consent and studied under institutional protocols 10-00329, 10-01272, and 10-02471, as approved by the Human Research Protection Program Committee on Human Research at the University of California, San Francisco.

### Tissue preparation and immunohistochemistry

Serial 5-μm sections were cut from paraffin-embedded tissue blocks. One section was stained with hematoxylin and eosin (H&E). Immunohistochemistry was performed on adjacent serial tissue sections by using standard protocols. Microwave antigen retrieval in citrate buffer was used for activin A, γH2AX, and CD31. Antigen retrieval was performed in EDTA for COX-2 and in citrate buffer for αSMA, in both cases for 60 minutes in an 80°C water bath.

The DCIS lesions were stained for activin A (Sigma-Aldrich, 1:120, St. Louis, MO), γH2AX (Upstate Biotechnology Lake Placid, NY, 1:150), COX-2 (Dako, Carpentaria, CA, 1:300), CD31 (Dako, Carpentaria, CA, 1:20), and αSMA (Dako,Carpentaria, CA, 1:6,400). Slides were scanned at 20× magnification by using an Aperio ScanScope Digital Slide Scanner (Aperio Technologies, Inc., Vista, CA). A minimum of five regions was chosen for qualitative assessment. Evaluation of activin A, γH2AX, COX-2, CD31, and αSMA staining intensity or the proportion of immune cell infiltrate (in H&E slides) was performed in a blinded fashion comparing the same regions. Staining intensity was scored as low to absent (low) or moderate to strong (high).

### Telomere-FISH

Telomere content was assessed by using telomere-specific FISH, as previously described [20]. In brief, slides were deparaffinized and rehydrated through a graded alcohol series, washed, and then steamed for 25 minutes. Genomic DNA was denatured for 2 minutes at 84°C. Two PNA probes, one specific for telomeres (Cy3-labeled, red) and one specific for centromeres (FITC-labeled, green), were hybridized to the tissues for 2 hours at room temperature. Slides were washed twice in 70% formamide, twice in PBS with 0.05% Tween-20, and thoroughly rinsed in deionized water before counterstaining the nuclei with DAPI. Tissues were air-dried and mounted with Prolong antifade reagent (Invitrogen, Grand Island, NY).

Tissues were imaged by using a Zeiss LSM 510 confocal microscope with a 63× objective. Serial slides, stained with H&E, were used to guide identification of the DCIS lesions. The absence of centromere signal was used to exclude regions of tissue or slides (three specimens) with poor fixation and inadequate PNA hybridization. Telomere lengths were qualitatively scored by visual assessment of two cellular compartments, DCIS epithelial cells and adjacent stromal cells, as previously described [20]. Lesions in which telomere signals were similar to or brighter than the adjacent stroma were scored as high. Lesions in which telomere signals were absent or less than the adjacent stroma were scored as low.

### Statistical methods

Two-sided *t *tests assuming unequal variance were used to test the relations between expression of each of several genes (*activin A, COX-2, HIF1α, VEGF, IL-6, IL-8, tenascin C, collagen 1A1, and fibronectin*) or proteins (activin A, IL-6, IL-8, and VEGF), prostaglandin or lactate levels in HMFs in co-culture, or exposed to activin A, PGE_2_, NS398, or DNA-damaging agents (etoposide, NU7026, or UVC). Changes in the expression for each gene or protein are shown relative to their respective control in each figure. Error bars represent the standard error of the mean, and statistical significance (*P *≤ 0.05) is denoted with an asterisk. A two-tailed Fisher Exact Test was used to evaluate the relation between staining intensity (high or low) for activin A and telomere FISH, γH2AX, COX-2, CD31, αSMA, or immune infiltrate. The Jmp 9.0 statistical package (SAS Institute) was used for all analyses.

## Results

Activation of a stress response in epithelial cells reprograms adjacent fibroblasts to produce proteins associated with desmoplasia

We demonstrated that telomere malfunction in mortal, nontumorigenic human mammary epithelial cells, with a compromised p16/Rb pathway (vHMEC), results in sustained induction of activin A [5]. Acting in a cell-extrinsic fashion, activin A induces cyclooxygenase-2 (COX-2) and its enzymatic byproducts, primarily prostaglandin E_2 _(PGE_2_), in adjacent epithelial cells, and activates protumorigenic epithelial phenotypes. To appreciate fully the impact of this novel stress response, we investigated whether mammary epithelial cells (vHMECs) with telomere malfunction, resulting in the production of activin A and PGE_2_, could induce phenotypes associated with desmoplasia in human mammary fibroblasts (HMFs).

**Figure 1:**
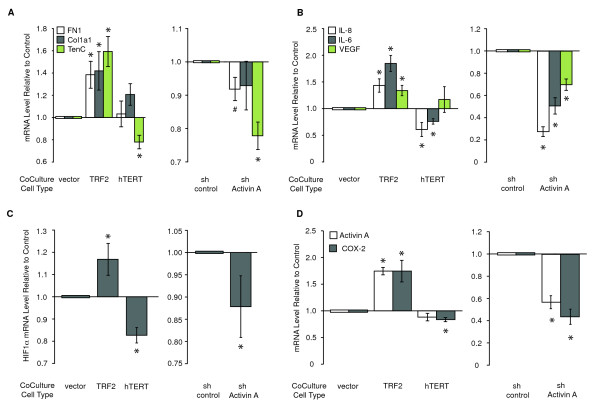
**Activation of a stress response in epithelial cells reprograms adjacent fibroblasts**. RM15 or RM21 human mammary fibroblasts (HMFs) were cocultured with RM78 or RM79 vHMECs for 24 hours in MEGM. vHMECs overexpressed either *TRF2, hTERT*, or pWP (vector control), or expressed a short hairpin for activin A (sh Activin A) or for luciferase (sh control). In all figures, error bars represent SEM, and asterisks denote statistical significance (*P *≤ 0.05) compared with appropriate control. Protein levels were measured with ELISA (duplicates), and mRNA levels were measured with Q-PCR (triplicate) and compared with appropriate controls (either vector or short hairpin). The relative levels of **(A) ***fibronectin (FN1), collagen 1A1 (Col1a1), tenascin C (TenC)*, **(B) ***IL-8, IL-6, VEGF*, **(C) ***HIF1α*, **(D) ***activin A*, and *COX-2 *mRNAs compared with either vector or short-hairpin controls for four HMF-vHMEC combinations are shown. In one instance (right side, panel A) only three HMF-vHMEC combinations were used. #, statistical significance (*P *≤ 0.05) compared with sh-control for this particular sample set.

To this end, we co-cultured HMFs with vHMECs overexpressing the telomere-binding protein *TRF2 *(TRF2-vHMEC) or vector control (pWP-vHMEC). We previously showed that *TRF2 *overexpression leads to telomere malfunction and triggers a double-strand DDR [5]. Three hallmarks of desmoplasia were assessed in the fibroblasts. We found an increased expression of ECM proteins (for example, *fibronectin (FN1), collagen 1A1 (Col1A1*), and *tenascin C (TenC)*; Figure 1A, left panel), elevated levels of selected cytokines (for example, proinflammatory interleukins *IL-8 *and *IL-6 *and proangiogenic vascular endothelial growth factor (*VEGF*); Figure 1B, left panel), and a switch to aerobic glycolysis [21], reflected by the induction of the transcription factor hypoxia-induced factor 1-alpha (*HIF1*α; Figure 1C, left panel) in HMFs co-cultured with TRF2-vHMECs. Similar to our previous findings in epithelial cells, reprogrammed HMFs exhibited an activation of the activin A pathway, as documented by increases in *activin A *and *COX-2 *transcript levels in HMFs co-cultured with TRF2-vHMEC (Figure 1D, left panel).

Having characterized the protumorigenic effects resulting from the overexpression of *TRF2 *in vHMECs, we investigated whether overexpression of the catalytic subunit of telomerase hTERT in vHMECs (hTERT-vHMEC) would have opposing effects for each of the documented phenotypes. In contrast to *TRF2*, *hTERT *maintains telomere function, represses the double-strand DDR, and decreases activin A expression in vHMECs [5]. As expected, many genes induced in HMFs co-cultured with TRF2-vHMECs (*tenascin C, IL-8 and IL-6, HIF1α*, and *COX-2*) were repressed in HMFs co-cultured with hTERT-vHMECs (Figure 1A through 1D, left panels).

### Activin A is necessary and sufficient to induce protumorigenic phenotypes in HMFs

To establish causality between the induction of activin A in epithelial cells and the production of desmoplasia-associated proteins in reprogrammed HMFs, we determined whether activin A was necessary for the induction of these genes by silencing its expression in vHMECs with a short-hairpin RNA to activin A (sh Activin A-vHMEC), as described [5]. HMF cells cocultured with vHMECs expressing a short hairpin to luciferase (sh control-vHMECs) were used as a baseline. Repression of *activin A *in vHMECs resulted in significant decreases in each of the previously measured end points, except *collagen 1a1*, which showed a nonstatistically significant decrease (Figure 1A through 1D, right panels). Thus, telomere malfunction in vHMECs induces many of the molecules associated with desmoplasia in neighboring fibroblasts via paracrine signaling. Additionally, we showed that *activin A *expression in vHMECs is necessary for the induction of these genes in HMFs.

**Figure 2:**
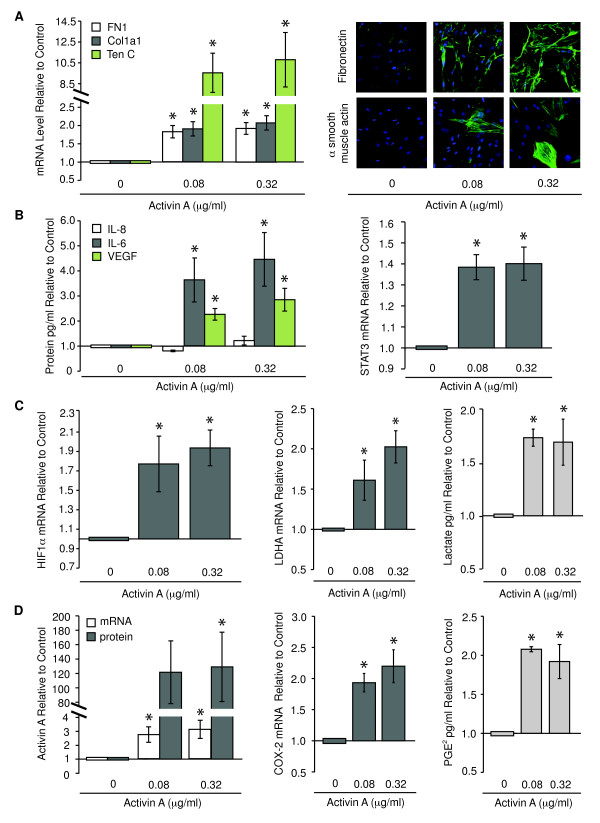
**Activin A is sufficient to induce genes and phenotypes consistent with desmoplasia in human mammary fibroblasts (HMFs)**. RM9, RM15, RM111, or RM124 HMF was exposed to exogenous activin A at the indicated doses for 48 hours. mRNA levels were assessed with Q-PCR. Cytokines, growth factors, and PGE_2 _were measured with enzyme-linked immunosorbent assay (ELISA). Lactate levels were evaluated with colorimetric assay. Mean expression changes for three individuals were analyzed in duplicate and expressed relative to control, **(A) ***fibronectin *(*FN1*), *collagen 1A1 *(*Col1a1*), and *tenascin C *(*TenC*) mRNA levels (left). Immunocytochemical detection of fibronectin or α-smooth muscle actin (αSMA, green) in RM156 HMF; nuclei in blue (right). **(B) **IL-8, IL-6, VEGF proteins (left) and *STAT3 *mRNA (right). **(C) ***HIF1α *mRNA (left), *LDHA *mRNA (middle), and lactate (right). **(D) ***Activin A *mRNA and protein (left), *COX-2 *mRNA (middle), and PGE_2 _levels (right).

To investigate whether exogenous activin A was sufficient to induce genes associated with desmoplasia in HMFs, we exposed HMFs to exogenous activin A for 48 hours. Two doses were tested to determine whether the response to activin A was dose dependent: one similar to the absolute amount induced by DNA damage (0.08 μg/ml), and the other, a fourfold greater dose (0.32 μg/ml). As observed in HMFs cocultured with TRF2-vHMECs, HMFs directly exposed to exogenous activin A exhibited an increase in the four groups of genes described in Figure 1. Increased *fibronectin, collagen 1A1*, and *tenascin C *mRNA levels (Figure 2A, left panel) and an accumulation of fibronectin and α-smooth muscle actin (αSMA) proteins (Figure 2A, right panel) were observed for stromal proteins. HMFs also displayed an increase in expression of selected cytokines, such as IL-6 and VEGF proteins (3.6- and 2.8-fold, respectively; Figure 2B, left panel) and their mutual downstream effector, signal transducer and activator of transcription 3 (*STAT3*; Figure 2B, right panel). Interestingly, although IL-8 protein and mRNA levels were increased by direct exposure to DNA damage (see later) and coculture with TRF2-vHMECs, respectively (Figure 1B, left panel), they were not changed on exposure to exogenous activin A (Figure 2B, left panel). HMFs exposed to activin A showed a switch to aerobic glycolysis with an increase in *HIF1α *(1.8-fold; Figure 2C, left panel) and one of its transcriptional targets known to drive the switch to aerobic glycolysis, lactate dehydrogenase A (*LDHA*; Figure 2C, center panel). The concomitant increase in lactate (Figure 2C, right panel), the product of LDHA, demonstrates the functional relevance of this finding.

Finally, consistent with our previous studies in vHMECs, activin A induced its own expression (Figure 2D, left panel), as well as that of *COX-2 *(Figure 2D, center panel) and the subsequent production of PGE_2 _in HMFs (Figure 2D, right panel).

We also evaluated the response of HMFs as a function of activin A dose for a subset of the genes described earlier. As shown in Additional file 2, *activin A *and *HIF1α *mRNAs were induced by as little as 0.005 μg/ml of exogenous activin A in HMFs. *Tenascin C *and *IL-6 *levels were increased in response to as little as 0.0012 μg/ml of activin A. Taken together, these observations showed that HMFs exhibited a dose-dependent response when directly exposed to activin A and that even very low concentrations of activin A could induce a desmoplastic-like signature.

### COX-2 activity is necessary and sufficient to induce protumorigenic phenotypes in HMFs

**Figure 3:**
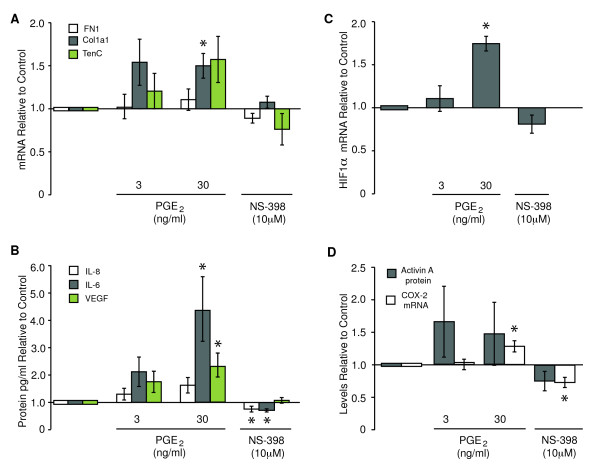
**COX-2 activity is sufficient to induce phenotypes consistent with desmoplasia**. RM9, RM15, RM111, or RM124 human mammary fibroblasts (HMFs) were exposed to exogenous PGE_2 _or the COX-2 inhibitor, NS398, for 48 hours. mRNA levels for *fibronectin (FN1), collagen 1A1 (Col1a1), tenascin C (TenC)*, and *HIF1α *were assayed in triplicate, and protein levels for IL-8, IL-6, VEGF, and activin A were analyzed in duplicate for at least three individuals. Relative levels of **(A) ***FN1, Col1a1, TenC*, **(B) **IL-8, IL-6, VEGF, **(C) ***HIF1α*, and **(D) ***activin A *and *COX-2 *were expressed relative to controls.

Is COX-2, acting downstream of activin A, responsible for reprogramming HMFs? One would predict that this is the case, because prostaglandins, which are products of COX-2 activity, can upregulate IL-8, IL-6, VEGF, and HIF1α in tumor epithelial cells [22,23]. To determine whether COX-2 activity was sufficient to trigger protumorigenic phenotypes in fibroblasts, we treated HMFs with two doses of PGE_2_, 3 and 30 ng/ml, similar to those measured in vHMECs with elevated COX-2 expression in response to telomere malfunction [5]. In HMFs treated with the high dose of PGE_2_, the levels of *fibronectin *remained unchanged, *collagen 1A1 *was moderately induced, and *tenascin C *expression increased, although this increase was not statistically significant (Figure 3A). HMFs also displayed increases in IL-8 (1.6-fold), IL-6 (4.2-fold), and VEGF (2.3-fold) proteins (Figure 3B) and *HIF1α *(1.7-fold) mRNA (Figure 3C) and modest increases in *activin A *protein and *COX-2 *mRNA (Figure 3D).

To determine whether COX-2 activity was necessary for the induction of these genes, we treated HMFs with 10 μ*M *COX-2 inhibitor NS398, a dose that ablated PGE_2 _levels by 70% (data not shown). Treatment of HMFs with NS398 resulted in a 25% and 30% decrease in IL-8 and IL-6 protein levels, respectively (Figure 3B), but had no effect on *fibronectin*, *collagen 1A1, tenascin C, VEGF, HIF1α*, or activin A (Figure 3A through 3D, respectively). *COX-2 *mRNA was repressed by NS398 and induced by PGE_2_, suggesting that COX-2 activity participated in a positive-feedback loop to drive its own expression in HMFs (Figure 3D). In summary, PGE_2 _induced *collagen 1A1*, IL-6, VEGF, *and HIF1α*. COX-2 activity was necessary only for the induction of IL-8 and IL-6. Collectively, these data suggest that COX-2 mediates the induction of some, but not all, of the molecules induced by activin A in HMFs and that these secreted factors may work in concert to elicit an extensive stromal reaction to epithelial stress signals.

### Conditioned media from HMFs exposed to activin A enhance motility of epithelial cells

**Figure 4:**
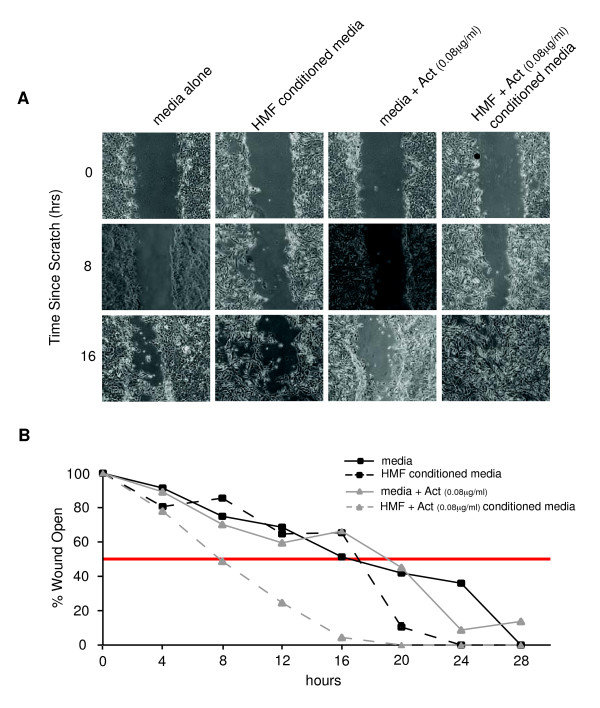
**Conditioned media from human mammary fibroblasts (HMFs) exposed to activin A enhance motility of epithelial cells**. RM111 and RM124 HMFs were grown in 5% serum for 24 hours and treated with activin A or vehicle. Medium was conditioned for 48 hours after addition of activin A. Conditioned medium or unconditioned medium containing activin A or vehicle was diluted 1:2 (vol/vol) in MEGM and added to RM15 vHMEC confluent monolayers. After 24 hours, monolayers were "wounded" with a pipette tip, and photographed at 4-hour intervals for 28 hours. **(A) **Representative images of vHMECs exposed to conditioned media from HMFs treated with activin A or vehicle, or unconditioned medium containing activin A or vehicle. **(B) **Kinetics of vHMEC "wound" closure on exposure to HMF-conditioned medium (dashed lines) or medium alone (solid lines). Representative trends of duplicate analyses in both HMFs are presented. Red line, 50% of "wound" open.

Having shown that exogenous activin A is sufficient to induce expression of a variety of signaling molecules in reprogrammed HMFs, we investigated whether secretion of some of these molecules could, in turn, affect epithelial cell phenotypes. Based on previous reports showing that activin A and prostaglandins promote cell motility [22,24-26], we assessed the motility of vHMECs by using a cell-wounding assay. Cells were exposed to media alone, media with activin A (0.08 μg/ml), or conditioned media from HMFs exposed (or not) to activin A (0.08 μg/ml). The vHMECs exposed to conditioned medium from HMFs treated with exogenous activin A were more motile than were vHMECs exposed to any of the three other media described earlier (Figure 4). These data demonstrated that HMFs exposed to activin A secreted factor(s) that induced a motility phenotype in vHMECs. Collectively, these data showed that a stress response in vHMECs (induced by telomere malfunction), and the resulting production of activin A, generated an activated (desmoplastic) stroma. In turn, the activated stroma, through production of additional secreted factors, elicited a protumorigenic epithelial phenotype (increased cell motility). Activation of epithelial motility in these cells could propagate the seeding and spreading of a protumorigenic field of tissue. Importantly, this bidirectional cellular communication occurs in primary (nontransformed) cells, suggesting that alterations in epithelium-fibroblast communication may occur early in tumorigenesis.

### Intrinsic DNA damage in HMFs elicits a subset of phenotypes associated with signaling from extrinsic DNA damage

**Figure 5:**
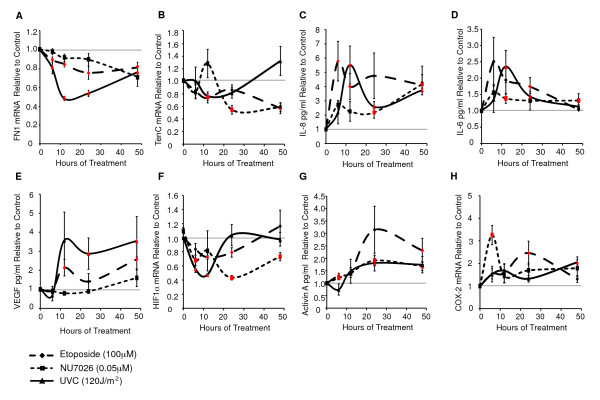
**Direct DNA damage in human mammary fibroblasts (HMFs) alters the expression of molecules associated with desmoplasia**. RM9, RM15, or RM156 HMFs treated with etoposide (double-stranded breaks), NU7026 (DNA-PK inhibitor), or UVC (single-stranded breaks) at the indicated doses. Average values (triplicates) for **(A) ***fibronectin (FN1)*, **(B) ***tenascin C (TenC)*, **(F) ***HIF1α*, and **(H) ***COX-2 *mRNAs or (duplicates) for **(C) **IL-8, **(D) **IL-6, **(E) **VEGF, and **(G) **activin A proteins are shown relative to untreated controls at each time point. The horizontal gray line indicates no change, and red markers show statistically significant differences compared with control.

We previously showed that double-strand DNA damage in epithelial cells results in an activin A-dependent induction of COX-2 [5]. Because exposure to DNA-damaging agents *in vivo *would affect not only epithelial cells, but also the entire tissue, we sought to determine whether activin A and COX-2 were induced in HMFs in response to cell-intrinsic DNA damage (Figure 5A through 5H). HMFs were exposed to etoposide (to induce double-strand DNA damage), NU7026 (a DNA-PK inhibitor that mimics telomere malfunction), and UVC (to induce single-strand DNA damage) by using the same conditions that induced activin A and COX-2 in vHMECs [5]. Interestingly, in contrast to the coculture experiments, cell-intrinsic DNA damage in HMFs repressed the expression of *fibronectin, tenascin C*, and *HIF1α *(Figure 5A, B, and 5F, respectively), three genes associated with desmoplasia that were induced by exogenous activin A (Figure 2A and 2C). Similar to our observations of fibroblasts cocultured with epithelial cells expressing a DDR, IL-8, IL-6, and VEGF proteins were induced in HMFs directly exposed to DNA-damaging agents (Figure 5C through 5E, respectively). Consistent with our previous findings in vHMECs [5], activin A and *COX-2 *were increased in HMFs exposed to all three agents, but to different extents and with different kinetics (Figure 5G and 5H).

Although HMFs exhibited very different toxicities to etoposide or NU7026 (4.2-fold versus 1.8-fold cell death after 48 hours; see Additional file 3), both exposures resulted in similar expression changes for most molecules investigated. Furthermore, treatment of HMFs with lower doses of etoposide (10 μ*M*) and UVC (60 J/m^2^), both of which induced a similar degree of cell death as NU7026 (0.05 μ*M*, Additional file 3), also induced the secreted factors IL-8, IL-6, and VEGF, activin A, and *COX-2 *(see Additional file 4B, C, D, F, and 4G, respectively). Likewise, *tenascin C *and *HIF1α *were repressed, even when cell death was low in HMFs treated with etoposide or UVC (see Additional file 4A and 4E). The findings that doses of stress agents that elicited minimal cell death still induced the expression of activin A, *COX-2*, and selected molecules associated with desmoplasia demonstrated that DNA damage *per se*, rather than cell death, accounted for these phenotypes.

### HMFs are less responsive to DNA damage-dependent induction of activin A than are vHMECs

**Figure 6:**
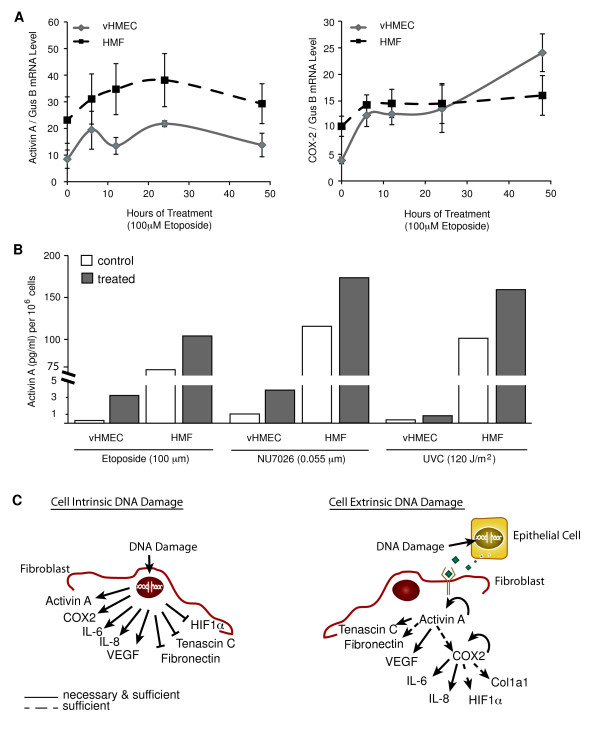
**Human mammary fibroblasts (HMFs) are less responsive to DNA damage-dependent induction of activin A than are vHMECs**. RM15 vHMEC and HMF, at the same passage, were treated with DNA-damaging agents (as described in Figure 5 legend). **(A**) *Activin A *and *COX-2 *mRNA levels were measured by using Q-PCR in triplicate and normalized to *GUSB*, used as an internal control, as described in the Methods section. **(B) **Activin A protein levels were measured with ELISA in duplicate 48 hours after treatment with the indicated agents. **(C) **Cartoon summarizing the expression changes after cell-intrinsic or cell-extrinsic DNA damage. Arrows indicate the induction of a gene or protein, whereas bars show repression of a gene or protein.

Because activin A and COX-2 were induced in both epithelial cells [5] and fibroblasts (Figure 5) in response to DNA damage, we compared the degree and timing of induction of these molecules in vHMECs and HMFs isolated from the same individual. HMFs had higher basal levels of *activin A *(2.7-fold) and *COX-2 *(2.6-fold) mRNAs than did vHMECs (Figure 6A). However, the fold induction of activin A was much less extensive in HMFs than in vHMECs exposed to damage (Figure 6B). In summary, the responses of vHMECs and HMFs to cell-intrinsic DNA damage vary in the magnitude of the induction of activin A and COX-2. Moreover, our observations suggest that the pathways induced in HMFs in response to cell-intrinsic DNA damage are not identical to those induced in response to exogenous activin A. More generally, these data imply that direct DNA damage to a tissue results in an accumulation of unique cell type-specific intrinsic and extrinsic responses (Figure 6C).

### Expression of activin A in DCIS is associated with reduced telomeres and desmoplastic-like phenotypes

Having characterized a complex stress response or stress-elicited extrinsic phenotype (SEEP) in epithelial cells and neighboring fibroblasts *in vitro*, we sought to validate the relevance of our findings *in vivo*. Our previous work showed that loss of telomere DNA is associated with a telomere-malfunction signature characterized by higher γH2AX (a DNA-damage marker), activin A, and COX-2 expression, in DCIS epithelia [5]. We used a pilot cohort of 16 DCIS cases (Table 2) to determine whether this telomere-malfunction signature was associated with an upregulation of phenotypes associated with desmoplasia. We assessed angiogenesis (CD31), the acquisition of "activated" fibroblasts (αSMA), and immune cell infiltration (visual inspection) adjacent to DCIS lesions. Telomere length was assessed with telomere-FISH in the 13 cases that were of sufficient quality.

**Figure 7:**
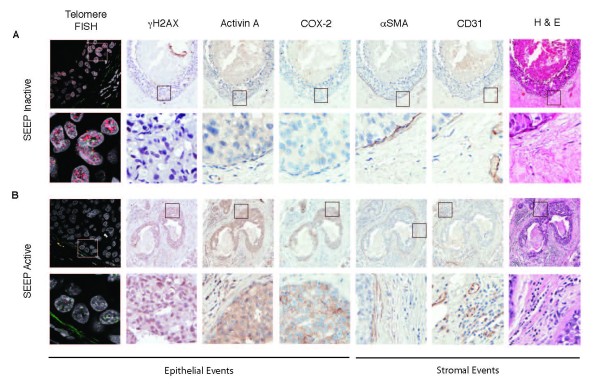
**Activin A in ductal carcinoma *in situ *is associated with reduced telomeres and desmoplastic-like phenotypes**. γH2AX, an indicator of DNA damage, activin A, COX-2, αSMA, and CD31 protein levels were assessed with immunohistochemistry (IHC) on serial sections of each DCIS lesion. Telomere signal (red) and centromere signal (green; internal control) in epithelial cells were compared with those in the adjacent stroma. Nuclei: DAPI (blue). Lesions were divided into two groups based on staining intensity for each protein and relative amounts of telomere signal (high or low) and of immune infiltrate (from H&E, high or low). **(A) **Stress-elicited extrinsic phenotype **(**SEEP) Inactive. Top row: serial sections of a representative DCIS lesion (10×) with high telomere-FISH signal, low expression of γH2AX, activin A, COX-2, αSMA, and CD31 proteins, and low immune infiltrate. Bottom row: 20× (IHC) or 63×-zoom (telomere-FISH) micrographs of the indicated regions shown in the top-row insets. **(B) **SEEP Active. Top row: serial sections of a representative DCIS lesion (10×) with low telomere-FISH signal, high expression of γH2AX, activin A, COX-2, αSMA, and CD31 proteins and high immune infiltrate. Bottom row: 20× (IHC) or 63×-zoom (telomere-FISH) micrographs of the indicated regions shown in the top-row insets.

Consistent with our previous study, we found that DCIS lesions with high activin A expression (Figure 7B) exhibited reduced telomere signal (*P *= 0.03) and higher levels of γH2AX (*P *= 0.01) and COX-2 (*P *= 0.01) when compared with lesions with low activin A (Figure 7A). Next, we interrogated whether activin A expression levels in a DCIS lesion could reflect the characteristics of adjacent stromal cells *in vivo*. DCIS lesions with high levels of activin A (Figure 7B) were associated with an increase in "activated" fibroblasts, as reflected by the increase in expression of αSMA (*P *= 0.01), angiogenesis, as illustrated by the increased expression of CD31 (*P *= 0.04), and immune cell infiltration (*P *= 0.007) in the adjacent stroma when compared with lesions with low levels of activin A (Figure 7A). These *in vivo *findings of stress-elicited extrinsic phenotypes (SEEPs), in the setting of preinvasive cancer, validate the conclusions drawn from our *in vitro *experiments (that is, high activin A levels in epithelial cells are associated with desmoplastic-like phenotypes in the adjacent stroma) (Table 3).

**Table 3:** High activin A expression in ductal carcinoma *in situ *(DCIS) is associated with stress-elicited extrinsic phenotypes

		**Telomere FISH^a^**		**γH2AX^b^**		**COX-2^b^**		**αSMA^b^**		**CD31^b^**		**Immune infiltrate^c^**	
	
	Low	1	6	6	2	7	1	7	1	6	2	6	2
	
Activin A	High	5	1	0	8	1	7	1	7	1	7	0	8
	
	*P *value	0.03		0.01		0.01		0.01		0.04		0.01	

^a^Telomere-specific fluorescence *in situ *hybridization (FISH) was evaluated on serial sections from each lesion. Lesions in which the telomere signals were equal or of greater intensity than adjacent stroma were scored as high. Lesions in which the telomere signal was less than the adjacent stroma were scored as low. ^b^Protein levels were evaluated by using immunohistochemistry (IHC) on serial sections from each lesion and scored as absent to low (low) or moderate to high (high). ^c^Immune infiltrate was evaluated on serial sections of each lesion stained with H&E and scored as absent to low (low) or moderate to high (high). *P *values were calculated by using a two-sided Fisher Exact Test.

## Discussion

These studies provide insights into early cell-cell interactions that participate in premalignancy and malignancy. They demonstrate that cell-intrinsic DNA damage can act beyond the cell in which the damage originally occurs and extend the consequences to reprogramming the neighboring epithelial and stromal cells in a dramatic and clinically relevant fashion. We previously showed that DNA damage and telomere malfunction in human mammary epithelial cells results in an activin A-dependent induction of COX-2 [5], causing cell-cycle arrest in non-p16-compromised epithelial cells and increased proliferation, motility, prostaglandin synthesis, and decreased apoptosis in p16-compromised epithelial cells [4,5]. We also established that secreted activin A can subsequently transmit a similar upregulation of COX-2 in adjacent epithelial cells that lack DNA damage or telomere malfunction. Thus, stress signals can be propagated beyond the cell with the original insult.

**Figure 8:**
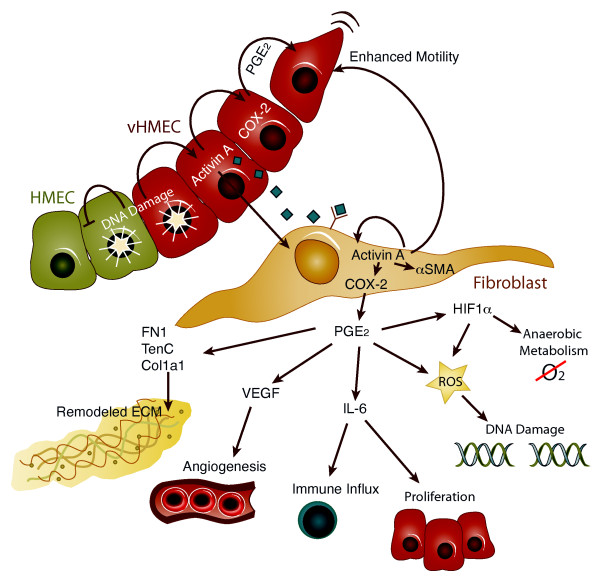
**Stress-elicited extrinsic phenotypes (SEEPs)**. In human mammary epithelial cells with an intact p16/Rb pathway (HMEC, green cells), DNA damage (or telomere malfunction) causes cell-cycle arrest and is self-limiting. In contrast, the same type of damage in human mammary epithelial cells with a compromised p16/Rb pathway (vHMEC, red cells) results in the activin A-dependent induction of COX-2 and continued cell proliferation [5]. Activin A can drive increased COX-2 expression in adjacent epithelial cells [5] and fibroblasts (tan cells; current work) via paracrine signaling. Fibroblasts adjacent to epithelial cells with telomere malfunction, or exposed to activin A or PGE_2_, upregulate a number of genes to induce cell-extrinsic phenotypes associated with a protumorigenic microenvironment, as shown [10,16]. Collectively, these factors can alter the extracellular matrix, induce angiogenesis, increase immune cell influx, drive cell proliferation, damage DNA, facilitate the switch to anaerobic metabolism, and promote cell motility [4,14,17,22,23,26,61-63].

Here we extend our initial observations by documenting that epithelial cells with DNA damage (telomere malfunction) can induce activin A and COX-2 in neighboring HMFs (Figures 1, 6B, 7, and 8). Moreover, activin A, functioning once again in a cell-extrinsic fashion, induces tumor-promoting phenotypes in HMFs and, presumably, in endothelial and immune cells. These phenotypes include altered expression and deposition of ECM proteins (fibronectin, αSMA collagen 1A1, and tenascin C), increased expression of several cytokines and growth factors (IL-8, IL-6, and VEGF), and a shift toward aerobic glycolysis (Figures 1 and 2). Furthermore, conditioned media obtained from HMFs exposed to activin A enhance the motility of epithelial cells, demonstrating that these epithelia-induced changes in HMFs can, in turn, alter the behavior of surrounding epithelial cells (Figure 4). These stress-elicited extrinsic phenotypes (SEEPs) document that early conversations between the epithelium and the stroma in carcinogenesis are truly bidirectional processes (Figure 8).

We hypothesize that these observations are critically relevant for tumor initiation and progression. DNA double-strand breaks (DSBs) (and the induction of DDR) are a common consequence of oncogene activation, replicative stress, inflammatory reactions, chromosomal breakage, and hypoxic stress, and also result from radio- and chemotherapy. Likewise, telomere malfunction occurs in virtually all tumor types, and, in many cases, in preinvasive lesions such as DCIS [27,28]. *In situ *hybridization has demonstrated that telomere malfunction typically occurs in the epithelial compartment [20,29] and is associated with poor prognosis and the progression of several malignancies [30-34]. The classic view is that DNA damage indirectly contributes to tumorigenesis through genomic instability that results in loss of tumor suppressors and activation of oncogenes. Our data suggest that these stress signals, acting in a cell-extrinsic fashion, can also directly contribute to tumorigenesis via early reprogramming to a protumorigenic stroma, even before the development of immortal tumorigenic epithelial cells.

Understanding how these signals contribute to cell-extrinsic events may provide novel insights critical for the detection, prevention, and treatment of cancer.

Additionally, our study shows that cellular stress in an epithelial cell can increase *HIF1α *levels, aerobic glycolysis, and lactate production in neighboring fibroblasts through secretion of activin A, a member of the TGF-β superfamily. The TGF-β pathway has been previously reported to drive increases in HIF1α, aerobic glycolysis, and lactate production in fibroblasts [35-37]. The mechanistic findings described by us are strikingly similar to those reported in the context of the Reverse Warburg Effect. In this latter phenotype, tumor epithelial cells induce an increase in aerobic glycolysis and lactate production in adjacent stromal cells through production of reactive oxygen species [21,38]. The lactate produced by stromal cells is, in turn, released in the local microenvironment, where it is used by tumor epithelial cells, presumably along with other metabolic intermediates, to fuel cell proliferation [14,37,38]. The novel stress response described by us here, and the Reverse Warburg Effect, therefore appear to be two programs that rely heavily on epithelium-fibroblast communication. In both cases, epithelial cells induce aerobic glycolysis in fibroblasts; such altered fibroblasts can then promote malignant phenotypes in epithelial cells (that is, motility or proliferation). These programs may therefore represent key complementary components of SEEPs.

The consequences of SEEPs may vary, depending on the cellular components of the tissue subjected to damage. DNA DSB or telomere malfunction in epithelial cells with intact p53 and p16 pathways would be contained, because we showed that overexpression of COX-2 in p16-competent HMECs activates cell-cycle arrest [5,6]. Likewise, cellular stress intrinsic to breast fibroblasts would also be relatively constrained because they arrest (data not shown) or die (see Additional file 3). In contrast, if the initiating damage occurs within an epithelial cell with a compromised p16 response (vHMEC), the consequences could be much more widespread. In these cells, overexpression of COX-2 is a proliferative signal and also causes an autoinduction of activin A and *COX-2*. The resultant secretion of factors on neighboring epithelial and stromal cells can be profound and everextending, both by virtue of paracrine signaling and by the induction of epithelial motility, which could expand the reprogrammed region. Strikingly, fibroblasts exposed to exogenous activin A do not arrest or die and additionally exhibit expression changes (that is, increased HIF1α, fibronectin, and tenascin C) that are not observed in fibroblasts with cell-intrinsic DNA damage.

We reported the presence of p16-compromised epithelial cells as expanded foci in healthy breast tissue [4,18]. Cells obtained from these foci have documented overexpression of COX-2 and telomere malfunction [4,7]. Premalignant lesions, such as DCIS, are often found in expanded foci of these cells *in vivo *[39]. Reaching a critical threshold of such cells could result in a sustained signaling that would remodel local tissue components, both epithelial and stromal, to support the synergistic emergence of a malignant lesion. In contrast, the presence of cells with intact p16 intermixed with cells that have activated SEEPs would limit SEEP signaling to a confined area and eventual extinction.

This view would be consistent with our previous studies demonstrating that DCISs with a compromised p16/pRB pathway and overexpression of COX-2 are at high risk for progressing to invasive cancer [6]. Likewise, DCIS lesions that exhibit a senescence signal, as indicated by increased p16 in the absence of proliferation, rarely progress [6]. Finally, in DCIS lesions, higher activin A expression is associated with telomere loss and increased COX-2 expression in the epithelial compartment, and increased expression of αSMA (which reflects activation of fibroblasts), increased immune cell infiltration, and increased angiogenesis in the adjacent stroma (Figure 7). These *in vivo *findings are consistent with the *in vitro *observations summarized earlier and provide a rationale for identifying biomarkers for risk stratification.

Some of the secreted molecules we describe have been reported as part of the secretory phenotypes associated with senescent cells [9,40]. The role of cellular senescence in malignancy is complex. The induction of senescence in epithelial cells constitutes a barrier to malignant transformation [41,42], perhaps through the induction of irreversible cell-cycle arrest in damaged cells at high risk for expansion and malignant progression. In keeping with this view, induction of senescence by DNA damage-inducing chemotherapy arrests tumor progression [43]. It has also been argued that factors secreted by senescent cells elicit deleterious cell nonautonomous effects that alter the tissue microenvironment [44]. Senescent fibroblasts secrete more than 40 factors associated with intercellular signaling, including IL-6 and IL-8, many of which have been implicated in tumor progression [8,45]. This complex phenotype, defined as senescence-associated secretory phenotype (SASP), is not a fixed phenotype but rather a secretory program with unique qualitative and quantitative functions depending on cell type, damage type, extent, and environmental conditions.

Several of the characteristics of SASPs are similar to those of SEEPs, in that both programs are triggered by DNA damage and upregulate a core of secretory proteins that exert protumorigenic phenotypes. However, SASPs can easily be distinguished from SEEPs. SASPs develop over several days, occurs only after DNA damage exceeds a threshold that is associated with irreversible cell-cycle arrest, and is amplified by the loss of p53 [44]. In contrast, SEEPs develop within hours, is triggered by levels of DNA DSBs that are compatible with cell proliferation, and finally, requires p53 activity [5].

Activin A, a lynchpin of the SEEP program, is a secreted factor and a member of the TGF-β superfamily. Like TGF-β, activin A functions in a cell type- and tissue-dependent fashion [46,47]. Studies evaluating the role of activin A in breast tumorigenesis have largely focused on the epithelial compartment, where activin A overexpression inhibits apoptosis and increases tumor volume in xenograft models of breast cancer [48]. Activin A is often upregulated in DCIS and in invasive breast cancer compared with normal breast tissue [49,50]. Higher activin A expression in various types of tumors with local recurrence or metastasis to lymph nodes supports that activin A may have prognostic significance [51,52] and help to predict response to neoadjuvant therapy [53].

The findings described here are also consistent with previous studies showing that activin A signaling plays an important role outside the epithelium. Activins mediate epithelial-stromal interactions during mammary gland branching [54]. High activin A levels in tumors are associated with upregulation of genes consistent with a desmoplastic stroma and progression to metastatic disease [53]. Secretion of activin A by carcinoma-associated fibroblasts (CAFs) enhances tumorigenesis [55]. Activin A serum levels are elevated in patients with metastatic breast tumors [56,57]. This demonstrates that exogenous activin A can induce a wide panel of molecules associated with protumorigenic phenotypes, and implies that, in addition to its role in epithelial cells, activin A may be a potent modulator of stroma structure and function.

The "activation" of fibroblasts mediated by SEEP provides a novel mechanism for initiation of a protumorigenic stromal response. CAFs are often the most abundant cell type within the protumorigenic or desmoplastic stroma, and logically directly contribute to acquisition of its characteristics [10,11,13]. Fibroblasts with CAF-like phenotypes have been postulated to derive from (a) resident fibroblasts, and/or (b) mesenchymal stem cells that have been progressively altered by exposure to tumor epithelial cells or their secreted factors [58-60]. In these scenarios, the generation of a CAF requires interaction with tumor epithelial cells, and therefore prior acquisition of tumorigenic phenotypes by the epithelial cell compartment. Importantly, our studies demonstrate that this conversation between epithelial and stromal cells occurs before tumorigenesis because the epithelial cells used in our study are mortal and nontumorigenic [7].

## Conclusions

In summary, we show that DNA damage (telomere malfunction) in mortal, nontumorigenic epithelial cells induces tumor-promoting phenotypes in adjacent HMFs through activin A and COX-2. Acting in a cell-extrinsic fashion, these molecules drive (a) increased expression and deposition of ECM proteins, (b) elevated levels of cytokines and growth factors, and (c) a shift toward aerobic glycolysis. Importantly, conditioned media from HMFs exposed to exogenous activin A enhance the motility of adjacent epithelial cells. Thus, the molecular conversation between the epithelia and stroma is truly bidirectional. This work extends our previous study, showing that activin A and COX-2, induced by DNA damage in epithelial cells, can alter the behavior of adjacent, unaffected epithelia [5]. Collectively, these stress-elicited extrinsic phenotypes (SEEPs) demonstrate that DNA damage has cell-extrinsic consequences that lead to reprogramming of both epithelial and stromal cells (Figure 8) and provide novel insights into the clinical implications of these early cell-cell interactions as they contribute to premalignancy and malignancy.

## Abbreviations

CAF: carcinoma-associated fibroblast; Col1A: collagen 1A1; COX-2: cyclooxygenase 2; DAPI: 4'-6-diamidino-2-phenylindole; DCIS: ductal carcinoma *in situ*; DDR: DNA-damage response; DNA-PK: DNA-dependent protein kinase; DSB: DNA double-strand break; ECM: extracellular matrix; ELISA: enzyme-linked immunosorbent assay; FBS: fetal bovine serum; FISH: fluorescence *in situ *hybridization; FITC: fluorescein isothiocyanate; FN1: fibronectin; γH2AX: gamma-histone 2AX; GUSB: β-glucuronidase; HIF1α: hypoxia-inducible factor 1α; HMF: human mammary fibroblast; HMEC: human mammary epithelial cell: intact p16/Rb pathway; hTERT: human telomerase reverse transcriptase; IL-6: interleukin 6; IL-8: interleukin 8; LDHA: lactate dehydrogenase A; PGE_2_: prostaglandin E_2_; PNA: peptide nucleic acid; Q-PCR: quantitative polymerase chain reaction; Rb: retinoblastoma protein; RM: reduction mammoplasty; SASP: senescence-associated secretory phenotype; SEEP: stress-elicited extrinsic phenotype; αSMA: α-smooth muscle actin; STAT3: signal transducer and activator of transcription 3; TenC: tenascin C; TGF-β: transforming growth factor β; TRF2: telomeric repeat-binding factor 2; UVC: ultraviolet subtype C; VEGF: vascular endothelial growth factor; vHMEC: variant human mammary epithelial cell: silenced p16 expression.

## Competing interests

The authors have no competing interests to disclose.

## Authors' contributions

CAF and TDT developed the experimental design. CAF, KTP, TBF, and RAD conducted and analyzed the *in vitro *experiments. ESH provided DCIS samples and patient history. JZ performed IHC staining. CAF and KTP performed telomere-specific FISH and qualitatively assessed the IHC staining and telomere-specific FISH. CAF, RAD, and TDT wrote the manuscript. All authors read and approved this manuscript for publication.

## Supplementary Material

Additional file 1 **Characterization of HMFs**. Representative HMFs (derived from three donors) and MCF7 mammary epithelial cells. The top row shows differential interference contrast (DIC) images of cell morphology. The cells were immunostained for a fibroblast-specific marker, fibronectin (middle row), and an epithelium-specific marker, E-cadherin (bottom row), both in red. Nuclei were visualized by using DAPI (blue). Click here for file

Additional file 2 **Dose response of HMFs treated with activin A on selected genes associated with desmoplasia**. RM111 HMFs were grown in the absence of serum for 24 hours and then exposed to exogenous activin A at the indicated doses for 48 hours. mRNA levels for each gene were assessed in triplicates with Q-PCR and normalized relative to GUSB, an internal control. Click here for file

Additional file 3 **Cell viability after treatment with DNA-damaging agents**. RM9, RM15, and RM156 HMFs were treated with etoposide, UVC, and NU7026 at the indicated doses and times. **(A) **The mean number of trypan blue-positive cells (an indicator of cell death) was expressed relative to untreated controls at each time point. **(B) **The mean number of total cells was expressed relative to untreated controls at each time point. Click here for file

Additional file 4 **Low doses of etoposide and UVC alter the expression of molecules associated with desmoplasia in HMF**. RM9, RM15, and RM156 HMFs were treated with 10 μ*M *etoposide (dashed line) or 60 J/m^2 ^UVC (solid line). Average values for protein levels (measured in duplicate) for IL-8 **(B)**, IL-6 **(C)**, VEGF **(D)**, and activin A **(F)**; mRNA levels (measured in triplicate) for Ten C **(A)**, HIF1α **(E)**, and COX-2 **(G) **are shown relative to untreated controls at each time point. Data points shown in red illustrate statistically significant differences. Click here for file
