# Supplementary figures and images for: Cell-extrinsic consequences of epithelial stress: activation of protumorigenic tissue phenotypes
Source: Breast Cancer Res. 2012 Dec 7;14(6):R155. doi: 10.1186/bcr3368 (PMC3786321; doi:10.1186/bcr3368)

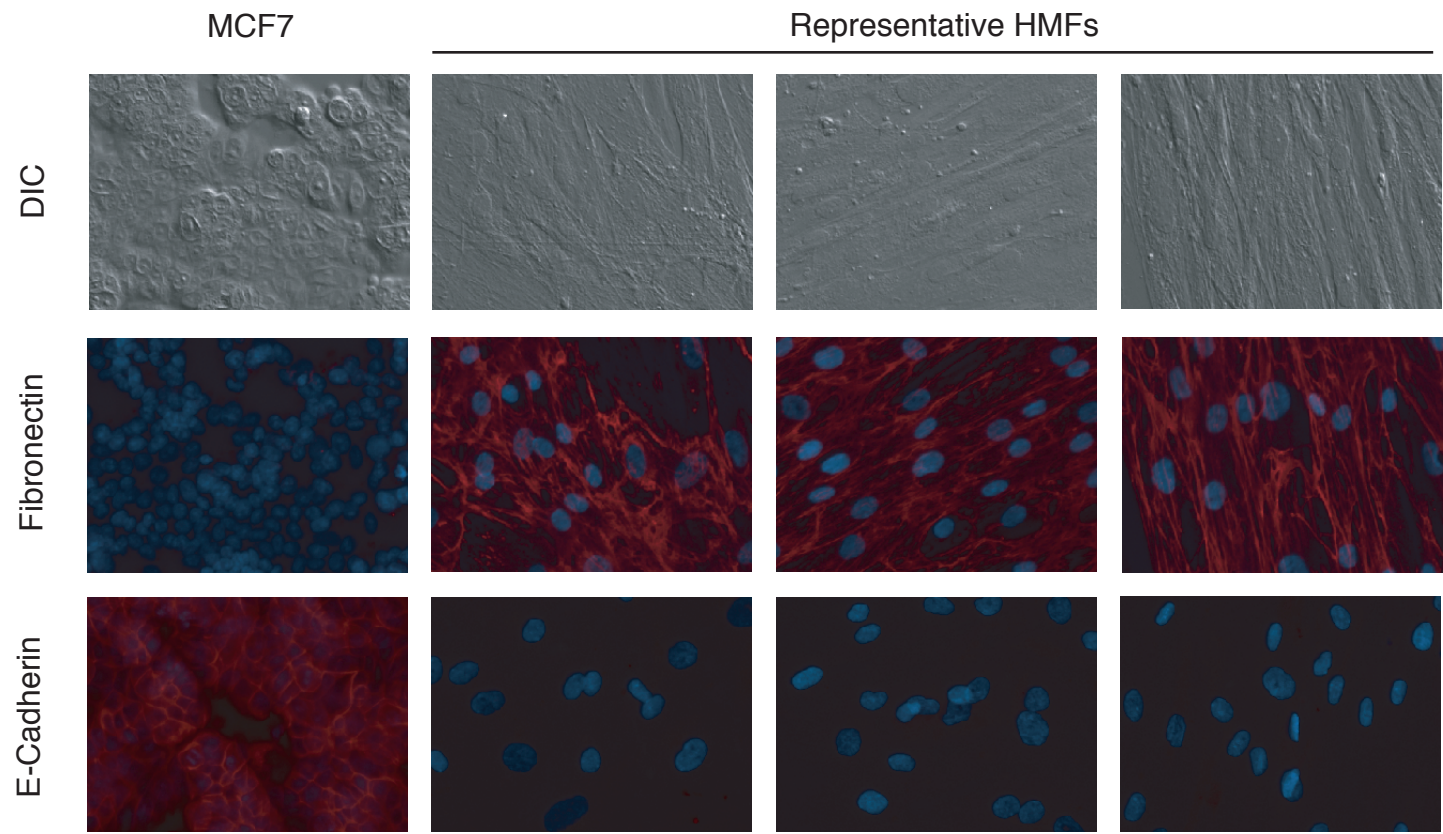

Supplement: Additional file 1 — Characterization of HMFs. Representative HMFs (derived from three donors) and MCF7 mammary epithelial cells. The top row shows differential interference contrast (DIC) images of cell morphology. The cells were immunostained for a fibroblast-specific marker, fibronectin (middle row), and an epithelium-specific marker, E-cadherin (bottom row), both in red. Nuclei were visualized by using DAPI (blue). [file bcr3368-S1.PDF]

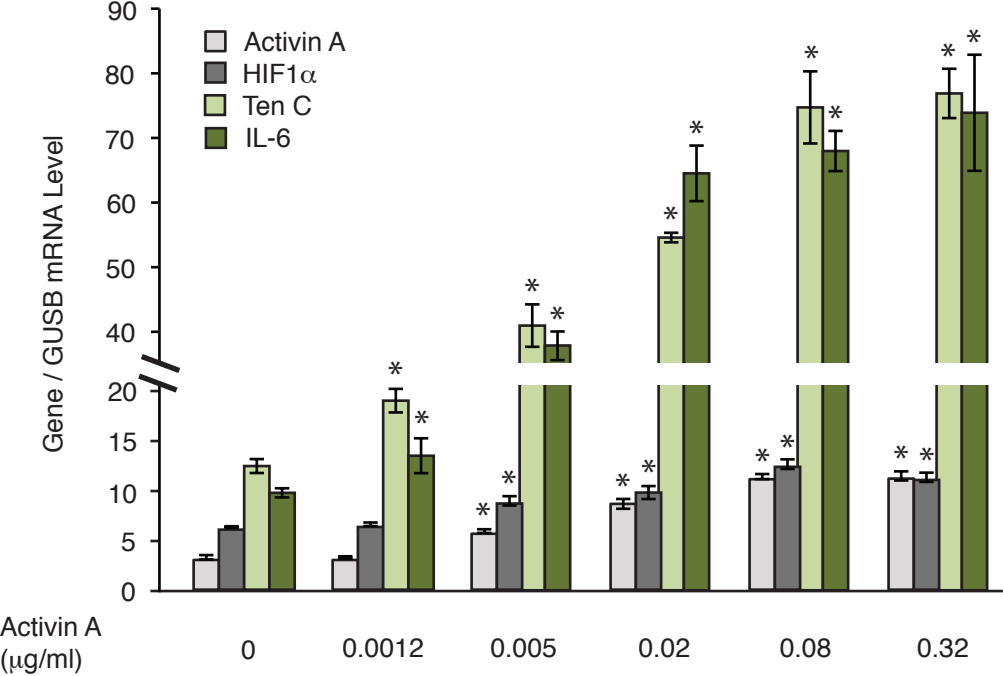

Supplement: Additional file 2 — Dose response of HMFs treated with activin A on selected genes associated with desmoplasia. RM111 HMFs were grown in the absence of serum for 24 hours and then exposed to exogenous activin A at the indicated doses for 48 hours. mRNA levels for each gene were assessed in triplicates with Q-PCR and normalized relative to GUSB, an internal control. [file bcr3368-S2.PDF]

**A**

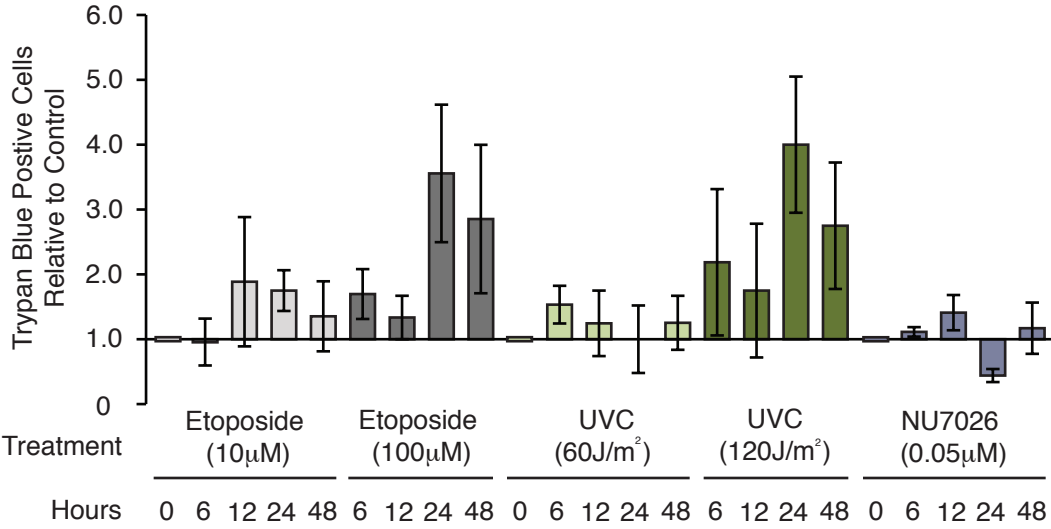

**B**

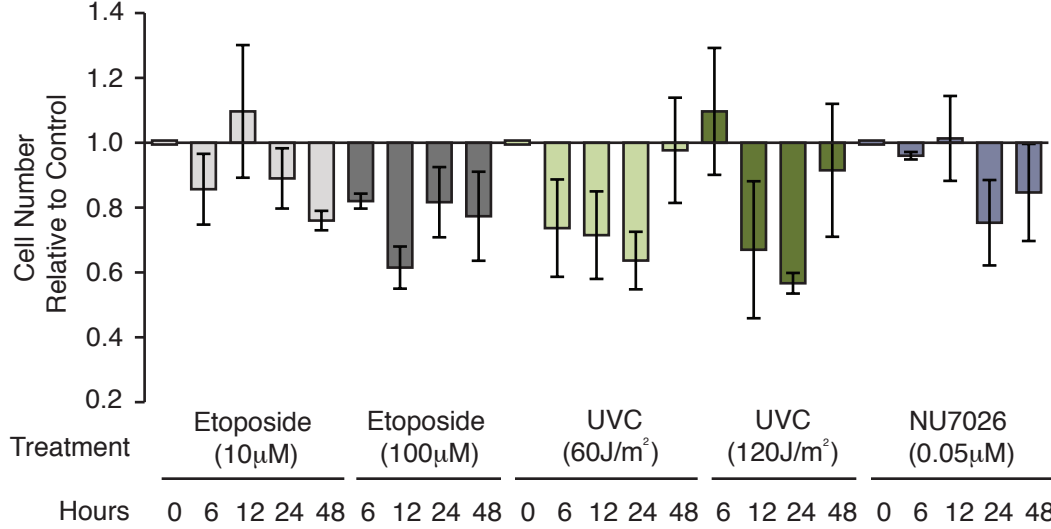

Supplement: Additional file 3 — Cell viability after treatment with DNA-damaging agents. RM9, RM15, and RM156 HMFs were treated with etoposide, UVC, and NU7026 at the indicated doses and times. (A) The mean number of trypan blue-positive cells (an indicator of cell death) was expressed relative to untreated controls at each time point. (B) The mean number of total cells was expressed relative to untreated controls at each time point. [file bcr3368-S3.PDF]

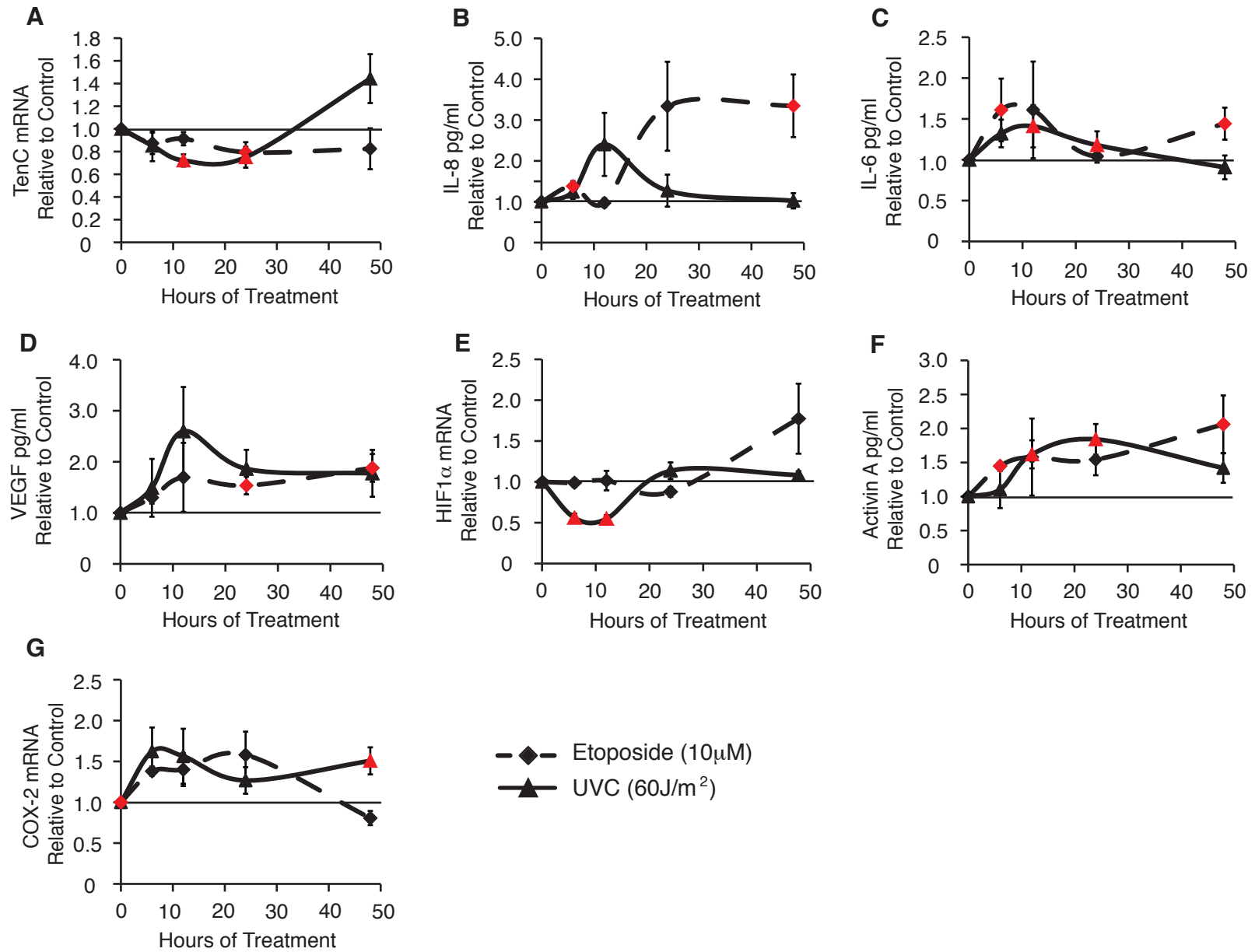

Supplement: Additional file 4 — Low doses of etoposide and UVC alter the expression of molecules associated with desmoplasia in HMF. RM9, RM15, and RM156 HMFs were treated with 10 μM etoposide (dashed line) or 60 J/m2 UVC (solid line). Average values for protein levels (measured in duplicate) for IL-8 (B), IL-6 (C), VEGF (D), and activin A (F); mRNA levels (measured in triplicate) for Ten C (A), HIF1α (E), and COX-2 (G) are shown relative to untreated controls at each time point. Data points shown in red illustrate statistically significant differences. [file bcr3368-S4.PDF]
